# Continuous relative phases of walking with an articulated passive ankle–foot prosthesis in individuals with a unilateral transfemoral and transtibial amputation: an explorative case–control study

**DOI:** 10.1186/s12938-023-01074-2

**Published:** 2023-02-15

**Authors:** Elke Lathouwers, Jean-Pierre Baeyens, Bruno Tassignon, Felipe Gomez, Pierre Cherelle, Romain Meeusen, Bram Vanderborght, Kevin De Pauw

**Affiliations:** 1 Human Physiology and Sports Physiotherapy Research Group, Vrije Universiteit Brussel, 1050 Brussels, Belgium; 2 Brussels Human Robotics Research Center (BruBotics), Vrije Universiteit Brussel, 1050 Brussels, Belgium; 3 Experimental Anatomy Research Group, Faculty of Physical Education and Physiotherapy, Vrije Universiteit Brussel, Brussels, Belgium; 4 THIM, Internationale Hochschule Fur Physiotherapîe, Landquart, Switzerland; 5 Faculty of Applied Engineering Sciences, Universiteit Antwerpen, Antwerp, Belgium; 6 Axiles Bionics, 1130 Brussels, Belgium; 7 Robotics Research Group, Vrije Universiteit Brussel and IMEC, Brussels, Belgium

**Keywords:** Lower-limb amputation, Prosthesis, Kinematics, Biomechanics

## Abstract

**Background:**

A mechanical ankle–foot prosthesis (Talaris Demonstrator) was developed to improve prosthetic gait in people with a lower-limb amputation. This study aims to evaluate the Talaris Demonstrator (TD) during level walking by mapping coordination patterns based on the sagittal continuous relative phase (CRP).

**Methods:**

Individuals with a unilateral transtibial amputation, transfemoral amputation and able-bodied individuals completed 6 minutes of treadmill walking in consecutive blocks of 2 minutes at self-selected (SS) speed, 75% SS speed and 125% SS speed. Lower extremity kinematics were captured and hip–knee and knee–ankle CRPs were calculated. Statistical non-parametric mapping was applied and statistical significance was set at 0.05.

**Results:**

The hip–knee CRP at 75% SS walking speed with the TD was larger in the amputated limb of participants with a transfemoral amputation compared to able-bodied individuals at the beginning and end of the gait cycle (*p* = 0.009). In people with a transtibial amputation, the knee–ankle CRP at SS and 125% SS walking speeds with the TD were smaller in the amputated limb at the beginning of the gait cycle compared to able-bodied individuals (*p* = 0.014 and *p* = 0.014, respectively). Additionally, no significant differences were found between both prostheses. However, visual interpretation indicates a potential advantage of the TD over the individual's current prosthesis.

**Conclusion:**

This study provides lower-limb coordination patterns in people with a lower-limb amputation and reveals a possible beneficial effect of the TD over the individuals’ current prosthesis. Future research should include a well-sampled investigation of the adaptation process combined with the prolonged effects of the TD.

## Introduction

Individuals with a transfemoral and transtibial amputation require an ankle–foot prosthesis to regain their ability to ambulate and to improve their quality of life [1]. However, wearing a passive ankle–foot prosthesis generates increased muscle activity of the intact limb and trunk, increased loading of the intact limb, damping, and increased trunk rotation which might cause tripping and falling [2, 3]. These gait dysfunctions can also initiate secondary injuries such as low back pain, muscle atrophy and osteoarthritis of the healthy knee and hip joints [4–7]. Such injuries entail high medical costs and lower the individuals' quality of life.

Development and evaluation of an ankle–foot prosthesis aim to mimic the ankle function of able-bodied individuals. In context, a major challenge is to increase ankle push-off power during walking [8, 9]. Unfortunately, passive prostheses cannot provide a sufficient range of motion and net positive joint work, which induces asymmetrical limb loading, altered gait and daily activity patterns compared to able-bodied individuals [9–15]. To improve prosthetic functioning, reduce injuries and improve quality of life, a new passive ankle–foot prosthesis (i.e. Talaris Demonstrator) has been developed. The Talaris Demonstrator (TD) originates from research with the Ankle Mimicking Prosthetic (AMP-) Foot [16–19] and is a prototype in the development process of an innovative passive ankle prosthesis (Lunaris) classified as energy storing and releasing foot with an ankle joint articulation allowing for plantar and dorsal flexion and with internal sensors allowing for the gathering of biomechanical data for predictive maintenance purposes [20]. “Its foot structure and leg structure are connected through the articulated ankle joint, allowing the composite spring element to store and progressively return energy during the complete stance phase. The leg structure connects to the shank through the male pyramid adaptor, and the prosthetic device is securely contained in the foot cover, with a Spectra sock.”

The prototype has been previously investigated in a cross-sectional study assessing functional performance through gait tasks (i.e. L-test, level walking, stair climbing, slope walking and backward walking) [20]. In this study, no clinically meaningful differences were found regarding performance, metabolic cost, heart rate, rating of perceived exertion, level of fatigue and comfort when comparing the TD with the individual's current prosthesis during gait tasks mentioned above. Nevertheless, a tendency towards increased comfort favouring the TD was found. Overall, these study results indicate that the TD performs equally well as the individuals’ current prostheses and that the TD shows indications of a potential benefit on comfort compared to the individuals’ current prostheses [20]. A biomechanical evaluation of TD is required to contextualise these results and further understand the walking patterns with the TD.

Walking patterns are frequently assessed by fundamental biomechanical measurements such as joint angles, moments and angular velocities [21]. Even though these outcome measures provide useful information and insights on gait patterns during prosthetic ambulation in individuals with transfemoral or transtibial amputation, a more advanced and holistic approach to investigating movement coordination encompasses the use of continuous relative phases (CRPs). This outcome measure is based on the dynamic systems theory explaining that movements are controlled in the neuromuscular system, generating signals to specific muscles and motor neurons [22, 23]. CRPs quantify the movement coordination between coupling segments or joints and allows for examining the stability and resilience to perturbation based on its variability while considering temporal and spatial parameters [22, 23]. More specifically, they distinguish movement patterns based on “in-phase” and “out-phase” behaviour [23]. During “in-phase” behaviour (considered a value of 0), the adjoining segments move in unison and rotate at an identical speed. During “out-of-phase” behaviour (considered a value of + 180° or − 180°), the adjoining segments move at identical speeds but in contrary directions to generate bending or twisting movements [23]. The positive and negative values for CRPs have also a qualitative meaning. If the phase angle of the proximal segment is subtracted from the phase angle of the distal segment, then positive continuous relative phase values indicate that the distal segment is ahead of the proximal segment in phase space and vice versa [23]. Using CRPs is beneficial to enhancing our understanding of the bearing impact of the adaptations following amputation at the level of coordination. However, its application is limited to people with unilateral transtibial or transfemoral amputation [24–29].

Given that the results found in terms of comfort when walking with the TD require further investigation, no biomedical evaluation of the TD has yet been conducted, and the literature on the use of CRPs in people with a lower-limb amputation is limited, this study aims to biomechanically evaluate the TD during level walking by mapping coordination patterns based on CRPs. More specifically, upon evaluating the TD, this study intends to differentiate the gait pattern of people with unilateral transfemoral and transtibial amputation compared to able-bodied individuals and aims to follow up with one individual with transtibial amputation over time to gain an exploratory assessment of the effect of the prosthesis.

## Results

### Participants’ characteristics

Seven participants with a lower-limb amputation (female = 2, male = 5) and 9 able-bodied individuals (female = 1, male = 8) completed the study protocol. Within the group of individuals with a lower-limb amputation, 4 had a TTA (female = 1, male = 3) and 3 a TFA (female = 1, male = 2). Among individuals with a TTA (right-sided amputation = 2), reasons for limb loss were trauma (*n* = 1), cancer (*n* = 1), medical error (*n* = 1) and congenital (*n* = 1). Limb loss within participants with a TFA (right-sided amputation = 6) was caused by trauma (*n* = 2) and cancer (*n* = 1). All individuals with a lower-limb amputation had a passive ankle–foot prosthesis. Participants’ characteristics are displayed in Table 1 and details regarding the individual characteristics of the participants with amputation are provided in Appendix A.1.

**Table 1:** Overview of participants’ characteristics displayed as mean ± standard deviation

	Participants with TTA (*n* = 4)	Participants with TFA (*n* = 3)	Able-bodied individuals (*n* = 9)
Age (years)	48.0 ± 15.9	59.3 ± 2.5	29.4 ± 5.7
Weight (kg)	85.8 ± 23.8	95.0 ± 17.3	82.3 ± 6.9
Height (cm)	176 ± 10.4	177.3 ± 11.0	183.2 ± 5.9
Residual limb length (cm)	16.9 ± 2.2	33.0 ± 6.6	Not applicable
Time since amputation (years)	4.0 ± 0.9	27.5 ± 17.0	Not applicable
Self-selected walking speed (km/h)	Current prosthesis: 4.5 ± 1.1	Current prosthesis: 2.3 ± 0.8	4.5 ± 1.8
	TD: 4.5 ± 1.1	TD: 2.5 ± 1.0	
Slow walking speed (km/h)	Current prosthesis: 3.4 ± 0.8	Current prosthesis: 1.7 ± 0.6	3.4 ± 1.3
	TD: 3.4 ± 0.8	TD: 1.9 ± 0.7	
Fast walking speed (km/h)	Current prosthesis: 5.6 ± 1.3	Current prosthesis: 2.9 ± 1.0	5.6 ± 2.2
	TD: 5.6 ± 1.3	TD: 3.1 ± 1.2	

Slow walking speed = 75% of the self-selected walking speed, fast walking speed = 125% of the self-selected walking speed, *TD*  Talaris Demonstrator

### Continuous relative phases across groups

**Fig. 1:**
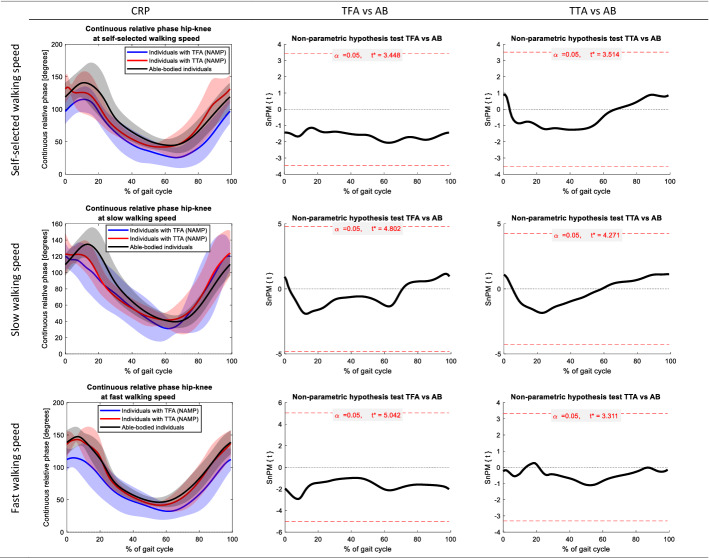
Continuous relative phases hip–knee of the non-amputated limb in people with a transfemoral amputation (TFA) and transtibial amputation (TTA) at self-selected, slow, and fast walking speed. Joint angles of able-bodied individuals (AB) are included as reference. The first column represents the mean (± SD) joint angle across all gait cycles. The gait cycles are defined based on the hip flexion peak angles. The second and third columns present the results of the non-parametric mapping (non-parametric independent t-tests) between TFA and AB, and TTA and AB, respectively. Red horizontal dashed lines depict the critical t-values

**Fig. 2:**
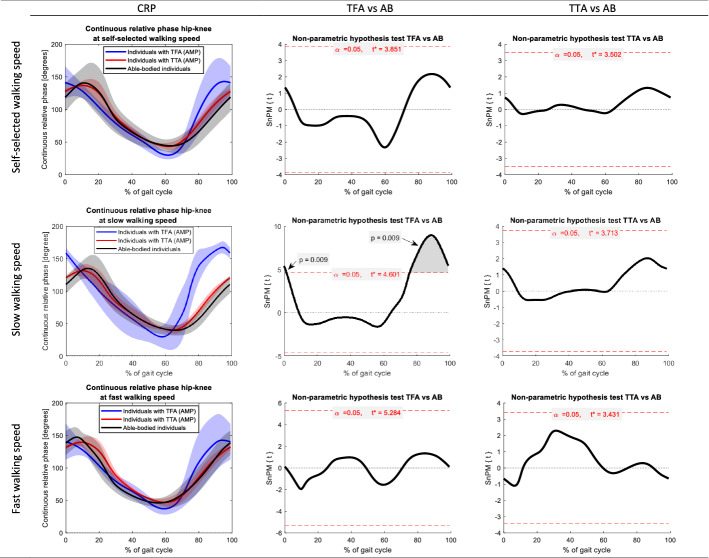
Continuous relative phases hip–knee of the amputated limb in people with a transfemoral amputation (TFA) and transtibial amputation (TTA) at self-selected, slow, and fast walking speed. Joint angles of able-bodied individuals (AB) are included as reference. The first column represents the mean (± SD) joint angle across all gait cycles. The gait cycles are defined based on the hip flexion peak angles. The second and third columns present the results of the non-parametric mapping (non-parametric independent t-tests) between TFA and AB, and TTA and AB, respectively. Red horizontal dashed lines depict the critical t-values

**Fig. 3:**
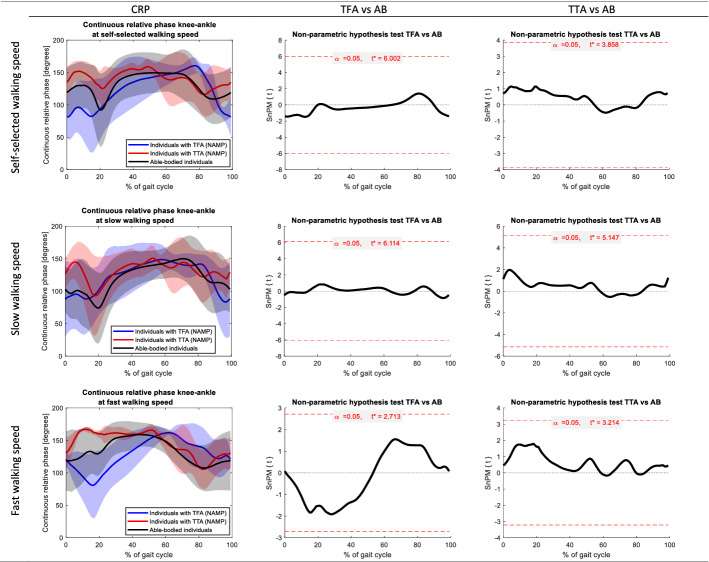
Continuous relative phases knee–ankle of the non-amputated limb in people with a transfemoral amputation (TFA) and transtibial amputation (TTA) at self-selected, slow, and fast walking speed. Joint angles of able-bodied individuals (AB) are included as reference. The first column represents the mean (± SD) joint angle across all gait cycles. The gait cycles are defined based on the hip flexion peak angles. The second and third columns present the results of the non-parametric mapping (non-parametric independent t-tests) between TFA and AB, and TTA and AB, respectively. Red horizontal dashed lines depict the critical t-values

**Fig. 4:**
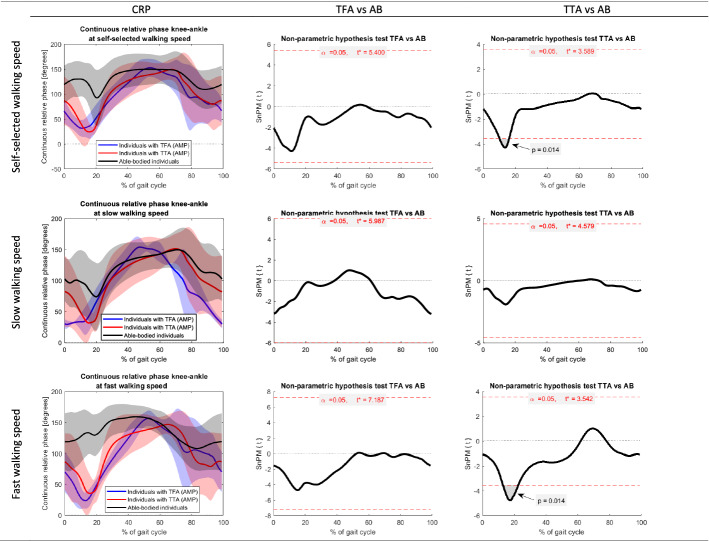
Continuous relative phases knee–ankle of the amputated limb in people with a transfemoral amputation (TFA) and transtibial amputation (TTA) at self-selected, slow, and fast walking speed. Joint angles of able-bodied individuals (AB) are included as reference. The first column represents the mean (± SD) joint angle across all gait cycles. The gait cycles are defined based on the hip flexion peak angles. The second and third columns present the results of the non-parametric mapping (non-parametric independent t-tests) between TFA and AB, and TTA and AB, respectively. Red horizontal dashed lines depict the critical t-values

Figures 1, 2, 3, 4 present the CRP hip–knee and knee–ankle of the sagittal plane while walking at self-selected speed, slow walking speed and fast walking speed with the TD. Statistical non-parametric mapping (SnPM) graphs (included in Figs. 1, 2, 3, 4) demonstrate the differences in CRPs between individuals with a TFA and able-bodied individuals, and between individuals with TTA and able-bodied individuals.

Across the CRPs hip–knee, we observed a significant difference at slow-speed walking between individuals with a TFA and able-bodied individuals (*p* < 0.05). The CRP hip–knee was significantly larger in the amputated limb of participants with a TFA at ± 0–5% (*p* = 0.009) and ± 75–100% (*p* = 0.009) of the gait cycle compared to able-bodied individuals. No other significant differences were found.

Across the CRPs knee–ankle, a significant difference was observed between the amputated limb of participants with a TTA and able-bodied individuals. The CRP knee–ankle in individuals with a TTA was smaller at ± 15–20% of the gait cycle during fast and self-selected walking speeds (*p* = 0.014 and *p* = 0.014, respectively) compared to able-bodied individuals. No other significant differences were found.

### Continuous relative phases across types of prosthesis

**Fig. 5:**
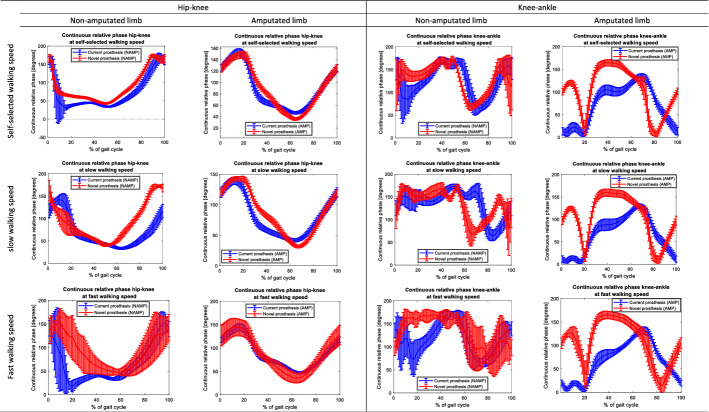
Comparison of continuous relative phases hip–knee and knee–ankle at self-selected, slow, and fast walking speed between current prosthesis and novel prosthesis (Talaris Demonstrator) without familiarisation (cross-sectional) in one individual with a transtibial amputation (ID01). The continuous relative phases shown in the first and third columns are those of the non-amputated limb (NAMP) and the ones in the second and last columns are those of the amputated limb (AMP). The continuous relative phases represent the mean continuous relative phase across all gait cycles and the error bars represent the standard deviation across all gait cycles. The gait cycles are defines based on the hip flexion peak angles

We explored the individual difference in CRPs between the current prosthesis and TD during walking. The CRPs did not differ between the current prosthesis and the TD within people with a TTA (Appendix B.1-B.3). Among participants with a TFA, no statistical tests could be conducted given the limited sample size (*n* = 3). Individual comparison across all individuals with lower-limb amputation appears to indicate closer correspondence and reduced standard deviations on the CRPs hip–knee and knee–ankle of the TD towards those of able-bodied individuals. As illustration, the individual plots of the CRPs of participant ID01 are provided in Fig. 5. The plots of participants ID2-ID7 are provided in appendix C.1-C.6.

**Fig. 6:**
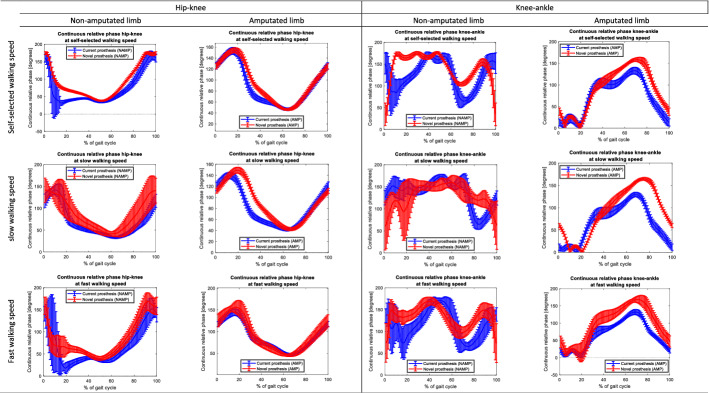
Comparison of continuous relative phases hip–knee and knee–ankle at self-selected, slow, and fast walking speed between current prosthesis and novel prosthesis (Talaris Demonstrator) after 42 days of familiarisation with the Talaris Demonstrator in one individual with a transtibial amputation (ID01). The continuous relative phases shown in the first and third columns are those of the non-amputated limb (NAMP) and the ones in the second and last columns are those of the amputated limb (AMP). The continuous relative phases represent the mean continuous relative phase across all gait cycles and the error bars represent the standard deviation across all gait cycles. The gait cycles are defines based on the hip flexion peak angle

Within this study, we monitored one participant with a unilateral transtibial amputation (i.e. ID01) over time while walking with the TD. The results in CRPs hip–knee and knee–ankle following the adaptation period of 42 days are depicted in Fig. 6. From an average perspective, we note that the CRPs correspond more closely to the gait pattern of able-bodied individuals.

## Discussion

This study aimed to biomechanically evaluate the TD during level walking by mapping inter-joint coordination patterns based on CRPs derived from the hip, knee and ankle joint angles (joint angles are available in appendix D.1, D.6).

When comparing walking with the TD in people with a TFA and TTA to able-bodied individuals, we found significant differences in the amputated limb in both groups (Figs. 1–4). Our results indicate a larger CRP hip–knee at slow-speed walking in the amputated limb of participants with a TFA while walking with the TD compared to able-bodied individuals at the beginning (± 0–5%) and end (± 75–100%) of the gait cycle (*p* = 0.009). Interpretation of these results and comparison with existing literature is hampered by the fact that we cannot specify the moment of heel strike and hence had to define the gait cycles differently. Considering these results, the hip angle during the swing phase is more prominent as it accounts for clearing the prosthetic foot from the ground during walking [30–32]. In people with a TTA, while walking with the TD, we found that the CRP knee–ankle in the amputated limb was smaller at ± 15–20% of the gait cycle during fast and self-selected walking speeds (*p* = 0.014 and *p* = 0.014, respectively) compared to able-bodied individuals. This difference could indicate the reduced knee–ankle coupling around heel strike [7]. The reduced knee–ankle coupling combined with the joint angles in people with transtibial amputation (appendix D.1 to D.6) suggests reduced knee extension and increased ankle dorsiflexion around 15–20% of the gait cycle. However, joint angles do not differ significantly from able-bodied individuals.

Previous studies reported increased variability in lumbopelvic, hip–knee and knee–ankle CRPs during walking in individuals with a transtibial amputation as opposed to a transfemoral amputation, as well as greater variability among individuals with an amputation relative to able-bodied individuals [24–29]. This increased variability results from the mitigation strategies to meet propulsion requirements and cope with the loss of function. The increased variability may reflect the high incidence of secondary injuries within the prosthetic population and may underlie the high incidence of falls [2, 3, 33, 34]. Our results concur with previously reported observations and shed a broader view on lower-limb coordination during walking in individuals with amputations. More specifically, this work's contribution to the current state-of-the-art consists of comparing hip–knee and knee–ankle CRPs between individuals with a TTA, TFA and able-bodied individuals and between the individuals' current prosthesis and the TD."

Upon comparing the CRPs of the TD and current prosthesis in individuals with TTA, no differences were found between the new and current prostheses (Appendix B.1-B.3). In individuals with a TFA, we did not conduct a non-parametric paired t-test due to the limited sample size. Based on these results combined with the previously published physiological evaluation of the TD, we can assume that the TD does not underperform as opposed to the individual's current prostheses during walking [20]. Additionally, from an individual perspective across all cases, there appears to be an advantage for the new prosthesis in correspondence of the CRPs hip–knee and knee–ankle towards those of able-bodied individuals and in reducing the standard deviations on these measurements (case-by-case plots are provided as supplementary materials). Part of our study’s novelty is that we could monitor one participant with a unilateral transtibial amputation over 42 days while walking with the TD (i.e. ID01 presented in Figs. 5 and 6). This follow-up allows some insights into the adaptation to a new prosthesis and can be of added value to the prosthetics community, as literature on adaptation processes to new prosthetic devices is scarce [35]. The visual interpretation suggests a potential advantage of the TD over the individual's current prosthesis during walking after 42 days. This indication is presumably attributable to the TD's articulated ankle joint. However, a larger cohort is needed to evaluate the TD to substantiate, along with a thorough comparison with any existing prosthetic devices featuring an articulated ankle joint.

Given the study's exploratory nature, the comparisons made, and the case–control study design with a sample size of 7 individuals with a lower-limb amputation (n_TTA_ = 4, n_TFA_ = 3), the reliability of our results is limited with a potential risk of statistical type 2 error. Furthermore, we could not control for age and time since amputation between all groups and could not provide participants with a 4-week familiarisation time to adapt to the TD. However, we matched for walking protocol resulting in a highly pragmatic trial with better external validity. The people with an amputation received one hour of familiarisation time before performing the walking test, which is inadequate to detect apparent differences in performance [36]. Conversely, we provided one individual with a TTA to use the device for 7 weeks (ID01), although the TD prototype is still under development. The adaptation period provides valuable insights into the functional performance of the TD. However, more people with an amputation should be included and monitored in time during their daily activities to understand the adaptation process to a new prosthesis, reproduce our current findings and evaluate the TD itself thoroughly. A final limitation concerns defining the gait cycles based on the hip-peak-flexion and predicting the stance and swing phases. Applying portable inertial measurement units in combination with foot-pad sensors in the future to determine CRPs can offer added value in terms of representativeness and insights towards rehabilitation and prevention by identifying the movement strategies of people with amputations while performing their daily activities beyond the clinical laboratory setting.

## Conclusion

The Talaris Demonstrator was developed to improve prosthetic gait in people with a lower-limb amputation. This study found no differences between the individuals’ current prosthesis and the TD and provides an overview of the lower-limb coordination patterns in people with a lower-limb amputation compared to able-bodied individuals through continuous relative phases. Nevertheless, this study reveals a possible beneficial effect of the Talaris Demonstrator for participants with a lower-limb amputation based on individual interpretation of the difference in continuous relative phases between the current prosthesis and the Talaris Demonstrator. Future research should include a well-sampled investigation of the adaptation process combined with the prolonged effects of the Talaris Demonstrator to evaluate our current findings.

## Materials and methods

### Participants

Participants with unilateral transfemoral and transtibial amputation (TFA and TTA, respectively) were recruited by contacting rehabilitation centres and orthopaedic departments of hospitals in Belgium and through social media between February and March 2022. All participants (aged 25–75 years) completed their rehabilitation and had a Medicare Functional Classification level K2-4. Adults with a bilateral, a trans-articular knee or hip, or additional upper limb amputation were excluded, as well as participants with neurological disorders, stump pains and wounds or with a bad socket fit. In addition to the participants with amputation, a control group of 9 able-bodied individuals was recruited via convenience sampling to enable comparison. All participants provided their written consent after being written and verbally informed regarding the study protocol. The study was executed in compliance with the Declaration of Helsinki [37] and was approved by the medical Ethics Committee of the University Hospital of the Vrije Universiteit Brussel (B.U.N. 143201526629) and by the Federal Agencies for Medicines and Health Products (FAGG/80M0860).

### Protocol and measurements

Participants visited the lab and completed 6 min of treadmill walking in consecutive blocks of 2 min. The consecutive blocks of 2 min consisted of walking at self-selected (SS) speed, at 75% of the SS speed (slow walking speed) and 125% of the SS speed (fast walking speed) [38]. The protocol was completed with the individual’s current prosthesis and the TD in a randomised order to enable comparison. Both devices were fitted to the individuals’ preferences by a prosthetist and participants performed the protocol when completely satisfied with the prosthetic alignment. The TD was fitted by adjusting the length of the pylon and pyramid connector and customising the amount of rigidity. All participants received a one-hour familiarisation period with the TD. After completing the protocol, one of the participants was allowed to use the TD for 7 weeks [36]. To explore the effect of adaptation to a new prosthetic device and unravel a possible benefit of the TD over the individuals’ current prosthesis, the protocol was repeated with the TD after the adaptation period of 7 weeks. During the walking tasks, lower extremity kinematics were captured and recorded continuously through wearable inertial measurement units that were placed bilateral on the thighs, legs and feet according to the manufacturer guidelines (Awinda, Xsens Technologies BV, The Netherlands) using MVN ANALYSE version 2021.2 (Xsens Technologies BV, The Netherlands).

### Data processing

The bilateral angles and velocities of hip, knee, and ankle joints for each individual per prosthetic condition were exported to Excel at SS speed, at 75% of SS speed and 125% of SS from MVN ANALYSE (version 2021.2). Each file contained 120 s of data. The files were truncated, and only the data from 30 to 100 s (approximately 65 gait cycles) were retained to reduce the file size. The excel files were then imported into a custom-written MATLAB script (version 2021a) to calculate the CRPs based on the phase angle and organise the kinematic data sets. Calculus of CRPs was based on the Hilbert transform [22, 23, 39].

### Statistical analyses

Statistical analyses were performed using MATLAB (version 2021a). Statistical non-parametric mapping (SnPM) was applied using non-parametric t-tests from the open-source spm1d-package (version 0.4.8, spm1d.org, ^©^ T. Pataky) to detect differences in joint angles and CRPs between individuals with a TFA and able-bodied individuals, and between TTA and able-bodied individuals. Joint angles are available in appendix D.1 to D.6. Statistical significance for the differences across groups was set at 0.05. Differences between the current prosthesis and TD were explored using SnPM paired t-tests. Due to the limited number of participants with a TTA (*n* = 4), the spm1d-package required a lower significance level of 0.1 to perform a SnPM paired t-test. Among participants with a TFA, no statistical tests could be conducted given the limited sample size (*n* = 3).

## Figures and Tables

**Fig. 7:**
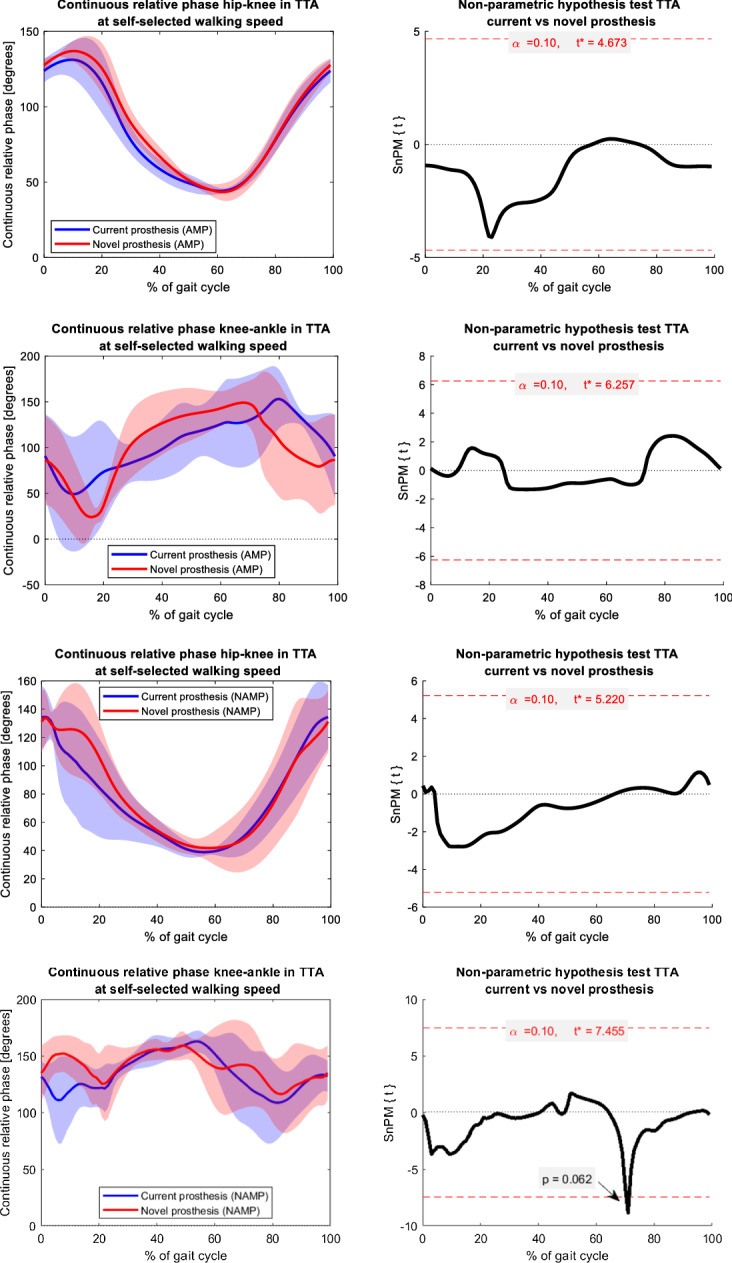
Comparison of continuous relative phases hip–knee and knee–ankle at self-selected walking speed between current prosthesis and novel prosthesis (Talaris Demonstrator) in individuals with a transtibial amputation (TTA). The continuous relative phases of the first and second row are those of the amputated limb (AMP) and the ones on the third and fourth row are those of the non-amputated limb (NAMP). The continuous relative hip–knee phases are displayed on rows one and three, and the continuous relative knee–ankle phases are presented on rows two and four. The continuous relative phases in the first column represent the mean (± SD) continuous relative phase across all gait cycles. The gait cycles are defined based on the hip flexion peak angles. The second column presents the results of the non-parametric mapping (non-parametric paired t-tests) between current prosthesis and Talaris Demonstrator. Red horizontal dashed lines depict the critical t-values

**Fig. 8:**
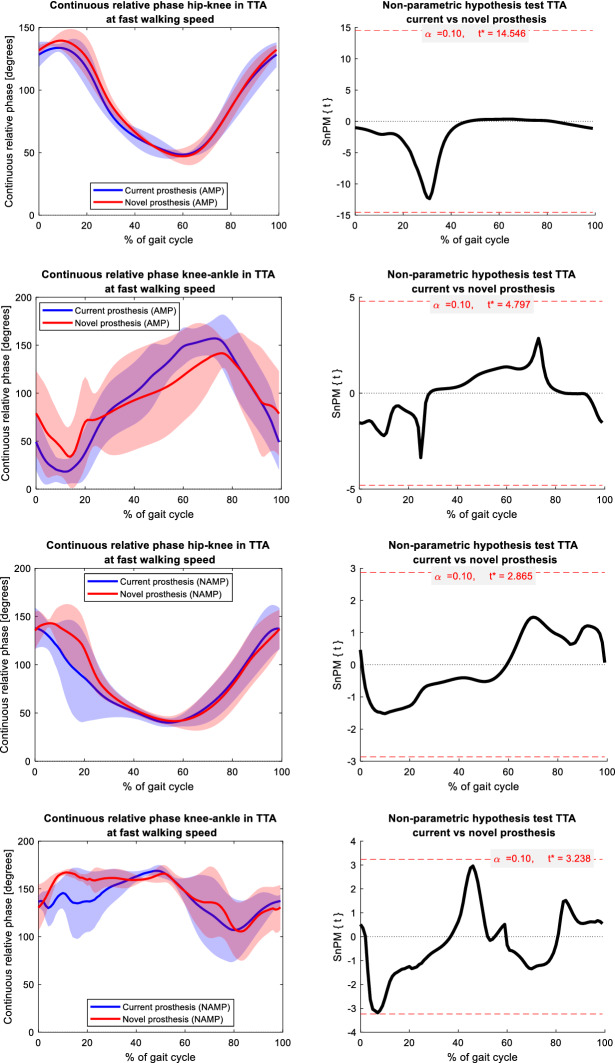
Comparison of continuous relative phases hip–knee and knee–ankle fast walking speed between current prosthesis and novel prosthesis (Talaris Demonstrator) in individuals with a transtibial amputation (TTA). The continuous relative phases of the first and second row are those of the amputated limb (AMP) and the ones on the third and fourth row are those of the non-amputated limb (NAMP). The continuous relative hip–knee phases are displayed on rows one and three, and the continuous relative knee–ankle phases are presented on rows two and four. The continuous relative phases in the first column represent the mean (± SD) continuous relative phase across all gait cycles. The gait cycles are defined based on the hip flexion peak angles. The second column presents the results of the non-parametric mapping (non-parametric paired t-tests) between current prosthesis and Talaris Demonstrator. Red horizontal dashed lines depict the critical t-values

**Fig. 9:**
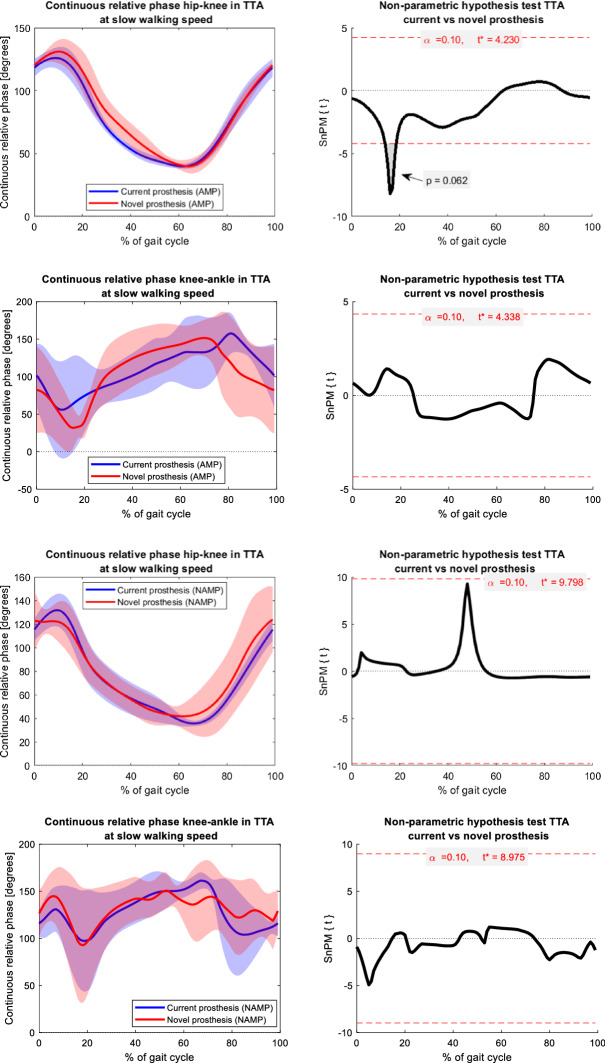
Comparison of continuous relative phases hip–knee and knee–ankle at self-selected walking speed between current prosthesis and novel prosthesis (Talaris Demonstrator) in individuals with a transtibial amputation (TTA). The continuous relative phases of the first and second row are those of the amputated limb (AMP) and the ones on the third and fourth row are those of the non-amputated limb (NAMP). The continuous relative hip–knee phases are displayed on rows one and three, and the continuous relative knee–ankle phases are presented on rows two and four. The continuous relative phases in the first column represent the mean (± SD) continuous relative phase across all gait cycles. The gait cycles are defined based on the hip flexion peak angles. The second column presents the results of the non-parametric mapping (non-parametric paired t-tests) between current prosthesis and Talaris Demonstrator. Red horizontal dashed lines depict the critical t-values

**Fig. 10:**
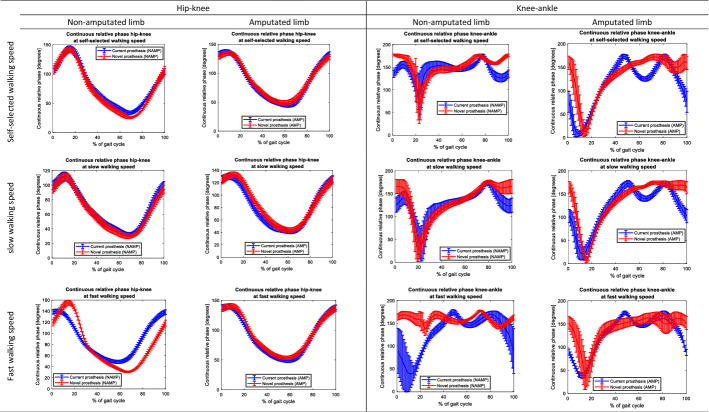
Comparison of continuous relative phases hip–knee and knee–ankle at self-selected, slow, and fast walking speed between current prosthesis and novel prosthesis (Talaris Demonstrator) in one individual with a transtibial amputation (ID02). The continuous relative phases shown in the first and third columns are those of the non-amputated limb (NAMP) and the ones in the second and last columns are those of the amputated limb (AMP). The continuous relative phases represent the mean continuous relative phase across all gait cycles and the error bars represent the standard deviation across all gait cycles. The gait cycles are defines based on the hip flexion peak angles

**Fig. 11:**
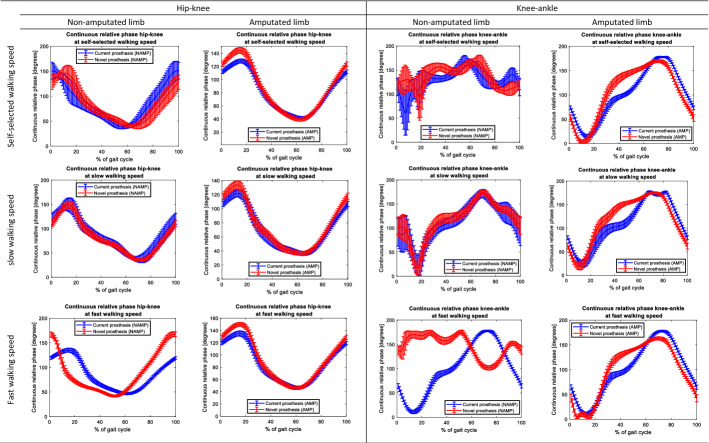
Comparison of continuous relative phases hip–knee and knee–ankle at self-selected, slow, and fast walking speed between current prosthesis and novel prosthesis (Talaris Demonstrator) in one individual with a transtibial amputation (ID03). The continuous relative phases shown in the first and third columns are those of the non-amputated limb (NAMP) and the ones in the second and last columns are those of the amputated limb (AMP). The continuous relative phases represent the mean continuous relative phase across all gait cycles and the error bars represent the standard deviation across all gait cycles. The gait cycles are defines based on the hip flexion peak angles

**Fig. 12:**
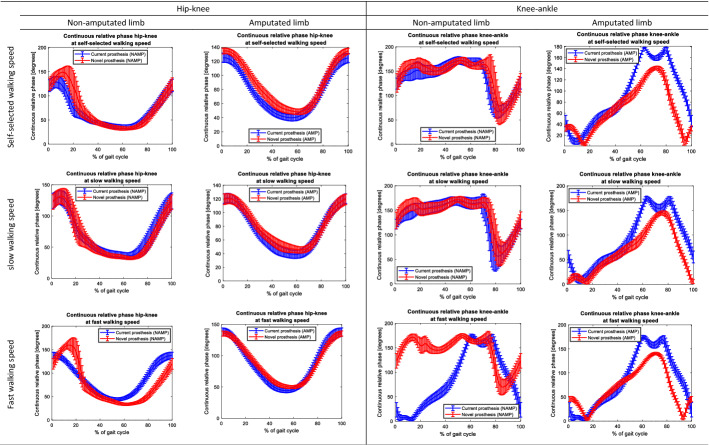
Comparison of continuous relative phases hip–knee and knee–ankle at self-selected, slow, and fast walking speed between current prosthesis and novel prosthesis (Talaris Demonstrator) in one individual with a transtibial amputation (ID04). The continuous relative phases shown in the first and third columns are those of the non-amputated limb (NAMP) and the ones in the second and last columns are those of the amputated limb (AMP). The continuous relative phases represent the mean continuous relative phase across all gait cycles and the error bars represent the standard deviation across all gait cycles. The gait cycles are defines based on the hip flexion peak angles

**Fig. 13:**
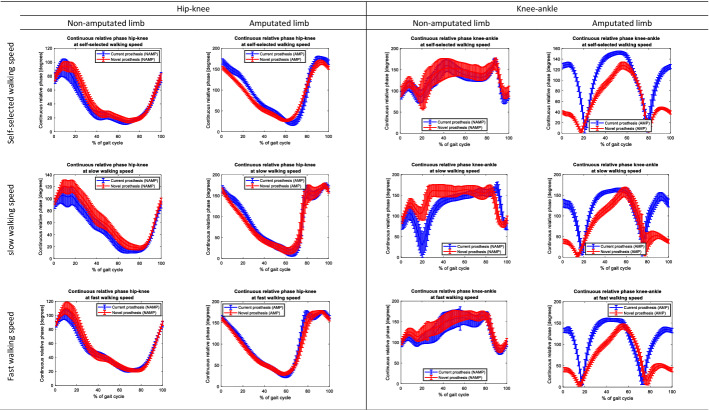
Comparison of continuous relative phases hip–knee and knee–ankle at self-selected, slow, and fast walking speed between current prosthesis and novel prosthesis (Talaris Demonstrator) in one individual with a transfemoral amputation (ID05). The continuous relative phases shown in the first and third columns are those of the non-amputated limb (NAMP) and the ones in the second and last columns are those of the amputated limb (AMP). The continuous relative phases represent the mean continuous relative phase across all gait cycles and the error bars represent the standard deviation across all gait cycles. The gait cycles are defines based on the hip flexion peak angles

**Fig. 14:**
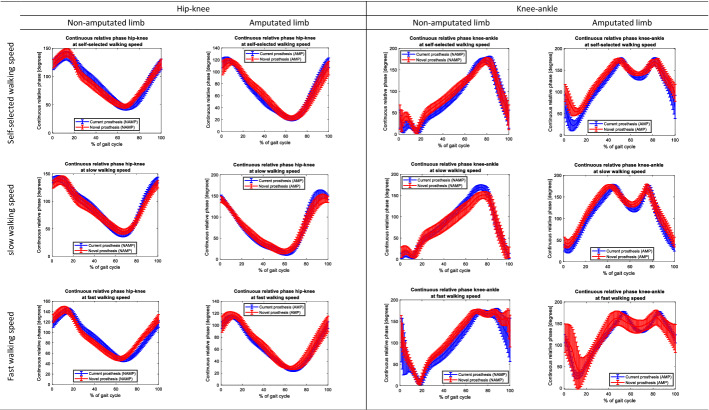
Comparison of continuous relative phases hip–knee and knee–ankle at self-selected, slow, and fast walking speed between current prosthesis and novel prosthesis (Talaris Demonstrator) in one individual with a transfemoral amputation (ID06). The continuous relative phases shown in the first and third columns are those of the non-amputated limb (NAMP) and the ones in the second and last columns are those of the amputated limb (AMP). The continuous relative phases represent the mean continuous relative phase across all gait cycles and the error bars represent the standard deviation across all gait cycles. The gait cycles are defines based on the hip flexion peak angles

**Fig. 15:**
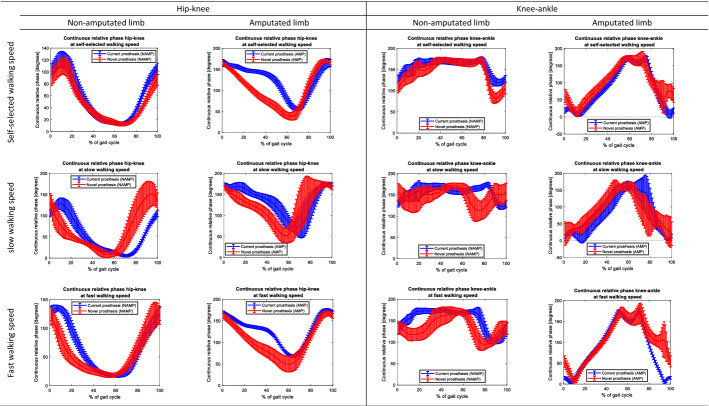
Comparison of continuous relative phases hip–knee and knee–ankle at self-selected, slow, and fast walking speed between current prosthesis and novel prosthesis (Talaris Demonstrator)) in one individual with a transfemoral amputation (ID07). The continuous relative phases shown in the first and third columns are those of the non-amputated limb (NAMP) and the ones in the second and last columns are those of the amputated limb (AMP). The continuous relative phases represent the mean continuous relative phase across all gait cycles and the error bars represent the standard deviation across all gait cycles. The gait cycles are defines based on the hip flexion peak angles

**Fig. 16:**
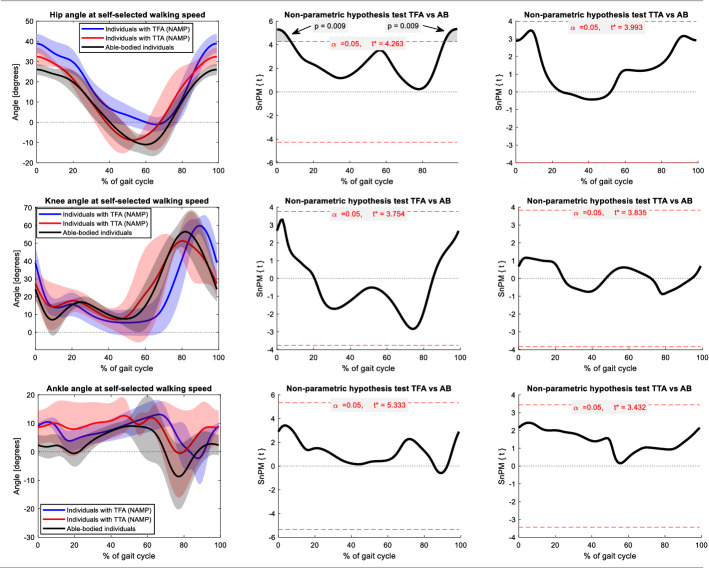
Joint angles at self-selected walking speed of the non-amputated limb in people with a transfemoral amputation (TFA) and transtibial amputation (TTA). Joint angles of able-bodied individuals (AB) are included as reference. The first column represents the mean (± SD) joint angle across all gait cycles. The gait cycles are defined based on the hip flexion peak angles. The second and third columns present the results of the non-parametric mapping (non-parametric independent t-tests) between TFA and AB, and TTA and AB, respectively. Red horizontal dashed lines depict the critical t-values

**Fig. 17:**
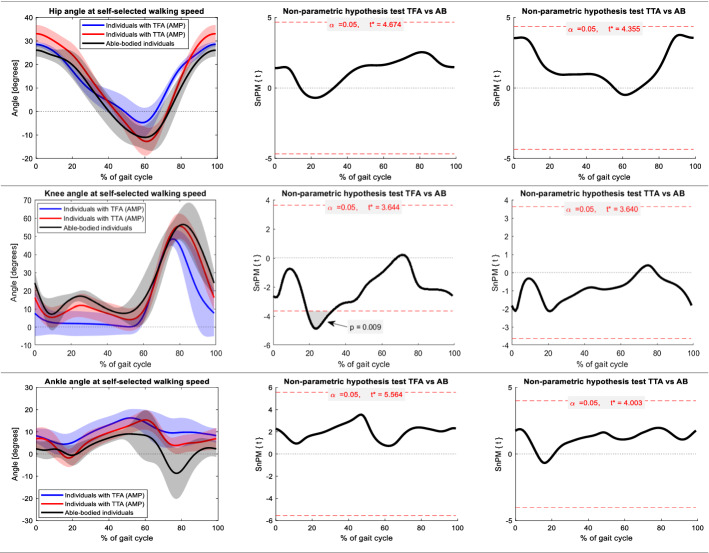
Joint angles at self-selected walking speed of the amputated limb in people with a transfemoral amputation (TFA) and transtibial amputation (TTA). Joint angles of able-bodied individuals (AB) are included as reference. The first column represents the mean (± SD) joint angle across all gait cycles. The gait cycles are defined based on the hip flexion peak angles. The second and third columns present the results of the non-parametric mapping (non-parametric independent t-tests) between TFA and AB, and TTA and AB, respectively. Red horizontal dashed lines depict the critical t-values

**Fig. 18:**
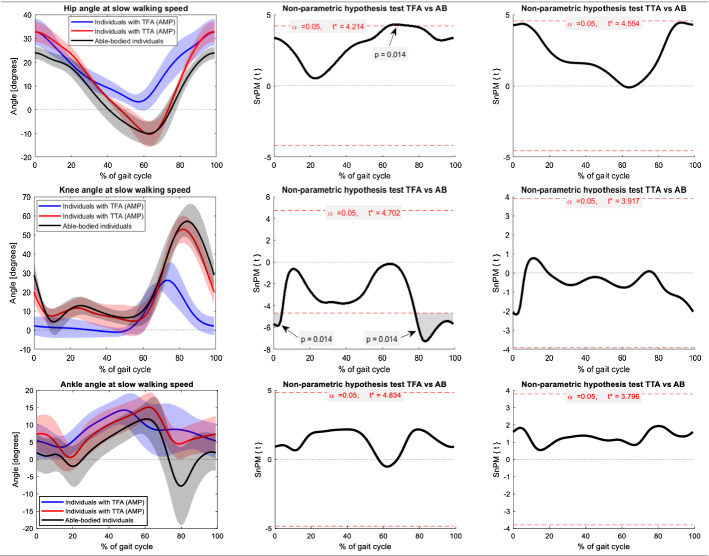
Joint angles at slow walking speed of the amputated limb in people with a transfemoral amputation (TFA) and transtibial amputation (TTA). Joint angles of able-bodied individuals (AB) are included as reference. The first column represents the mean (± SD) joint angle across all gait cycles. The gait cycles are defined based on the hip flexion peak angles. The second and third columns present the results of the non-parametric mapping (non-parametric independent t-tests) between TFA and AB, and TTA and AB, respectively. Red horizontal dashed lines depict the critical t-values

**Fig. 19:**
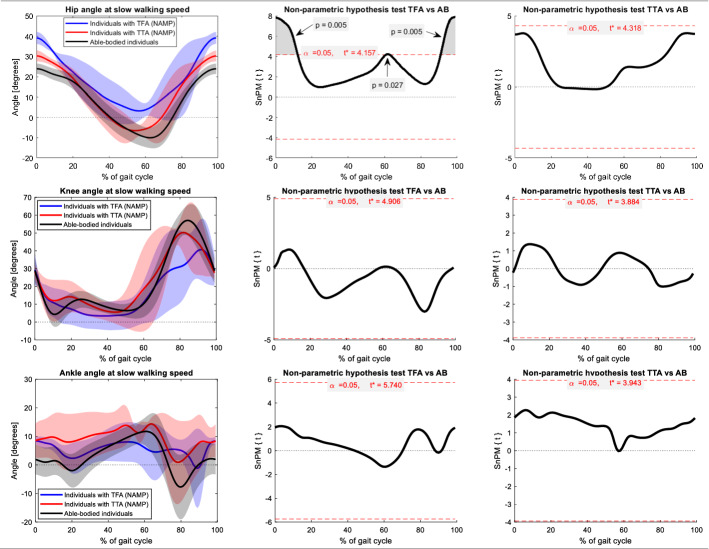
Joint angles at slow walking speed of the non-amputated limb in people with a transfemoral amputation (TFA) and transtibial amputation (TTA). Joint angles of able-bodied individuals (AB) are included as reference. The first column represents the mean (± SD) joint angle across all gait cycles. The gait cycles are defined based on the hip flexion peak angles. The second and third columns present the results of the non-parametric mapping (non-parametric independent t-tests) between TFA and AB, and TTA and AB, respectively. Red horizontal dashed lines depict the critical t-values

**Fig. 20:**
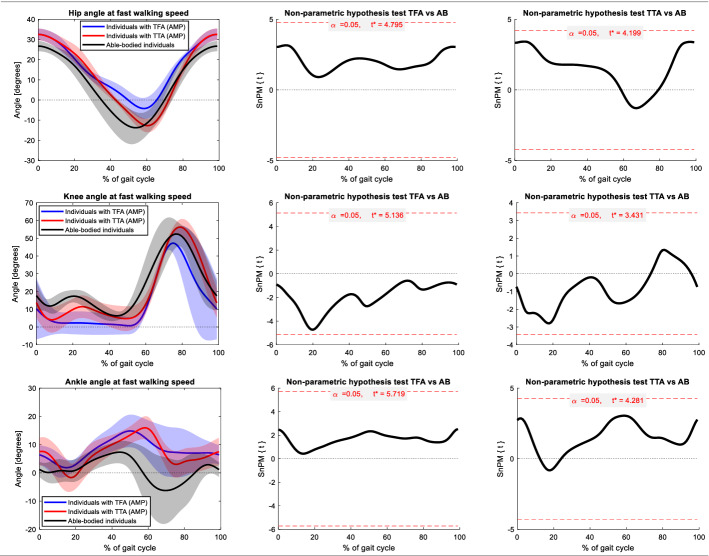
Joint angles at fast walking speed of the amputated limb in people with a transfemoral amputation (TFA) and transtibial amputation (TTA). Joint angles of able-bodied individuals (AB) are included as reference. The first column represents the mean (± SD) joint angle across all gait cycles. The gait cycles are defined based on the hip flexion peak angles. The second and third columns present the results of the non-parametric mapping (non-parametric independent t-tests) between TFA and AB, and TTA and AB, respectively. Red horizontal dashed lines depict the critical t-values

**Fig. 21:**
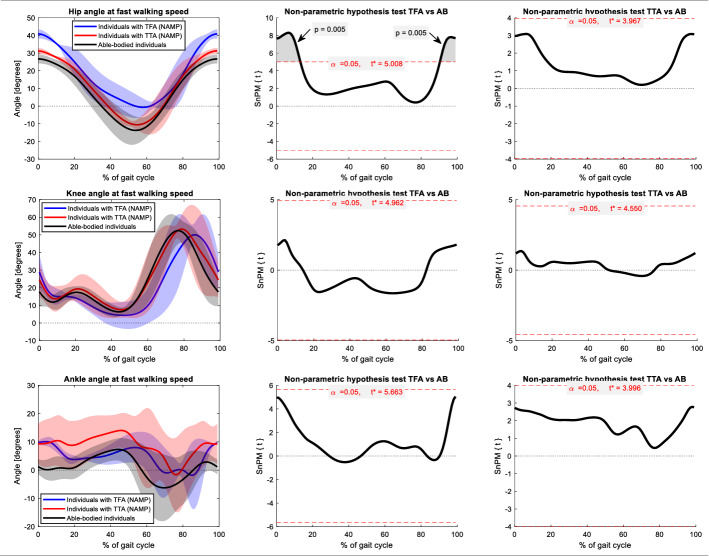
Joint angles at fast walking speed of the non-amputated limb in people with a transfemoral amputation (TFA) and transtibial amputation (TTA). Joint angles of able-bodied individuals (AB) are included as reference. The first column represents the mean (± SD) joint angle across all gait cycles. The gait cycles are defined based on the hip flexion peak angles. The second and third columns present the results of the non-parametric mapping (non-parametric independent t-tests) between TFA and AB, and TTA and AB, respectively. Red horizontal dashed lines depict the critical t-values

## Data Availability

The authors declare that the data supporting the findings of this study are available within this article. The processed kinematic datasets that support the findings of this study are available from the corresponding author, upon reasonable request.

## Appendix A

See Table 2

**Table 2:** Individual characteristics of the participants with a unilateral lower-limb amputation

Participant	Level of amputation	Current ankle–foot prosthesis	Knee prosthesis	Sex	Age (years)	Weight (kg)	Height (cm)	Prosthetic side	Stump length (cm)	Time since amputation (years)	Self-selected walking speed current prosthesis (km/h)	Self-selected walking speed novel prosthesis (km/h)
ID01	TTA	RUSH Foot HiPro	Not applicable	M	60	73	171	Left	15.0	3.5	4.0	4.0
ID02	TTA	RUSH Foot HiPro	Not applicable	M	38	94	182	Left	16.0	3.5	4.2	4.2
ID03	TTA	Össur LP Align	Not applicable	F	63	61	164	Right	16.5	5.0	3.6	3.6
ID04	TTA	Össur Pro-flex^®^ XC	Not applicable	M	31	115	187	Right	20.0	Not applicable	1.5	1.7
ID05	TFA	OTTOBOCK 3R80	Single axis knee	F	59	75	165	Right	26.0	36.4	3.1	3.6
ID06	TFA	OTTOBOCK Triton	OTTOBOCK Genium	M	57	105	181	Left	39.0	8.0	2.4	2.2
ID07	TFA	Össur Pro-flex^®^ LP	OTTOBOCK C-leg	M	62	105	186	Right	38.2	38.2	6.0	6.0

TTA participant with a unilateral transtibial amputation, TFA participant with a unilateral transfemoral amputation

## Differences in continuous relative phases between current prosthesis and Talaris Demonstrator

See Figures. 7, 8, 9

## Individual comparison of continuous relative phases hip–knee and knee–ankle at self-selected, slow, and fast walking speed between current prosthesis and novel prosthesis (Talaris Demonstrator) in individuals ID02–ID07.

See Figures. 10, 11, 12, 13, 14, 15

## Hip, knee, and ankle joint angles

Figures 16, 17, 18, 19, 20, 21 show the hip, knee and ankle sagittal joint angles while walking at self-selected speed, slow walking speed and fast walking speed with the Talaris Demonstrator. Statistical parametric mapping graphs (included in Figs. 1, 2, 3, 4, 5, 6) demonstrate the differences in joint angles between individuals with a transfemoral amputation and able-bodied individuals and between individuals with transtibial amputation and able-bodied individuals. No differences were found among hip, knee, and ankle joint angles between participants with a TTA and able-bodied individuals.

In participants with a TFA, we found that the hip joint angles of the non-amputated limb were significantly larger across the different walking speeds compared to able-bodied individuals. At self-selected speed, this difference was observed at 0–10% (*p* = 0.009) and above 90% of the gait cycle (*p* = 0.009). At slow walking speed, this difference occurred between 0 and 15%, at 60% and above 90% of the gait cycle (*p* = 0.005, 0.027, 0.005, respectively), and at fast walking speed, between 0 and 15% (*p* = 0.005) and above 90% (*p* = 0.005) of the gait cycle. Additionally, we found that the knee angle of the amputated limb was significantly smaller at 20–30% of the gait cycle compared to able-bodied individuals (*p* = 0.009).

## Abbreviations

TTA: Individual with a unilateral transtibial amputation
TFA: Individual with a unilateral transfemoral amputation
TD: Talaris Demonstrator
CRP: Continuous relative phase
SnPM: Statistical non-parametric mapping
SS: Self-selected speed
